# Polysaccharide and extracts from *Lentinula edodes*: structural features and antiviral activity

**DOI:** 10.1186/1743-422X-9-37

**Published:** 2012-02-15

**Authors:** Vinicius Pires Rincão, Kristie Aimi Yamamoto, Nágila Maria Pontes Silva Ricardo, Sandra Aguiar Soares, Luzia Doretto Paccola Meirelles, Carlos Nozawa, Rosa Elisa Carvalho Linhares

**Affiliations:** 1 Departamento de Microbiologia, Universidade Estadual de Londrina, Rod. Celso Garcia Cid., km 380, CEP: 86051-990, Londrina, Paraná, Brazil; 2 Departamento de Biologia Geral, Universidade Estadual de Londrina, Rod. Celso Garcia Cid., km 380, CEP: 86051-990, Londrina, Paraná, Brazil; 3 Departamento de Química Orgânica e Inorgânica, Universidade Federal do Ceará, Av. da Universidade, 2853, CEP: 60455-760, Fortaleza, Ceará, Brazil

**Keywords:** Lentinula edodes, Antiviral activity, Poliovirus, Bovine herpesvirus

## Abstract

**Background:**

*Lentinula edodes*, known as shiitake, has been utilized as food, as well as, in popular medicine, moreover, compounds isolated from its mycelium and fruiting body have shown several therapeutic properties. The aim of this study was to determine the antiviral activity of aqueous (AqE) and ethanol (EtOHE) extracts and polysaccharide (LeP) from *Lentinula edodes *in the replication of poliovirus type 1 (PV-1) and bovine herpes virus type 1 (BoHV-1).

**Methods:**

The time-of-addition assay was performed at the times -2, -1, 0, 1 and 2 h of the infection. The virucidal activity and the inhibition of viral adsorption were also evaluated. Plaque assay was used to monitor antiviral activity throughout.

**Results:**

The AqE and LeP were more effective when added at 0 h of infection, however, EtOHE was more effective at the times 1 h and 2 h of the infection. AqE, EtOHE and LeP showed low virucidal activity, and the inhibition of viral adsorption was not significant.

**Conclusions:**

The results allowed us to conclude that AqE, EtOHE and LeP act on the initial processes of the replication of both strains of virus.

## Background

Currently, there are a little more than 40 drugs approved for clinical use in the treatment of viral infections [1]. Studies regarding these drugs concentrated on synthetic products up to the end of the 80 s, when natural compounds attracted attention because of their efficacy in the inhibition of viruses important for human and animal health [2]. Natural products are recognized by the pharmaceutical industry because of their wide structural diversity, as well as, variety of pharmacological activities [3]. Amongst the sources of natural compounds, the fungi, especially the basidiomycetes, have stimulated interest from investigators.

The basidiomycete *Lentinula edodes *(Berkeley) Pegler (*Lentinus edodes*), known as shiitake, an edible mushroom native of the East Asia, is valued for its nutritional and medicinal properties and culinary and industrial applications [4]. Shiitake is the second most popular in the world [5] and in the last decades various compounds with therapeutic properties have been isolated from its mycelium and fruiting body. Some of these have been widely studied, for example, the polysaccharide lentinan which has demonstrated high immunopotentiating and antimetastasic activities [6,7], antitumor activity [8,9], antibacterial, antifungal and antidiabetic activities [10,11], among others. However, few works have examined the antiviral activity of this basidiomycete [12-14].

Poliovirus is a non-enveloped virus with an icosahedral capsid symmetry, and a genome consisting of a positive single-stranded RNA. The virion is classified in the genus *Enterovirus*, belonging to the family *Picornaviridae*, which includes many other pathogens of great importance to humans and other animals [15]. Despite the efforts to eradicate the virus, there were 1352 reported cases of poliomyelitis in African and Asian countries in 2010 [16]. Bovine herpesvirus (BoHV) is an important pathogen for the cattle industry. Viron belongs to the subfamily *Alphaherpesvirinae*, family *Herpesviridae *[17], has a genome consisting of a linear double-stranded DNA within an icosahedral capsid, enclosed by an envelope. In order to reduce losses caused by BoHV, vaccines consisting of attenuated virus are being utilized with positive results. However, the control of infections is still difficult, due to the latency established by the virus after primary infection or after vaccination [18].

The basidiomycetes show various biological activities and low toxicity what make them a promising source of bioactive molecules. Therefore, the aim of this study was to determine the antiviral activity of aqueous and ethanol extracts and polysaccharide of *Lentinula edodes *in the replication of poliovirus and bovine herpesvirus.

## Methods

### Cells and virus

HEp-2 cell cultures (human larynx epithelial cells carcinoma--ATCC, CCL-23) were grown in Dulbecco's Modified Eagle Medium (DMEM) (Gibco-BRL, EUA*), supplemented with 10% fetal bovine serum (*) and 2 mM glutamine (Sigma Chem. Co., EUA**), 100 μg/ml streptomycin (**), 100 IU/ml penicillin (**) and 2.5 μg/ml amphotericin B (Bristol Myers-Squibb, Brazil).

The poliovirus type 1 (PV-1), vaccinal strain, was obtained from the ATCC (ATCC, VR-58) and the bovine herpesvirus type 1 (BoHV-1) was supplied by DMVP-UEL, Brazil. Both strains were propagated in HEp-2 cells, and virus titers determined by plaque assay.

### Aqueous and ethanol extracts

The aqueous extract (AqE) of *Lentinula edodes *(lineage IW) was obtained as follows. Ground basideocarp was resuspended with distilled water, heated at 60°C for 1 h and centrifuged at 3000 × g for 5 min. The supernatant was pre-filtered and submitted to ultrafiltration in 0.2 μm pore size membrane, and stored at -20°C.

The ethanol extract (EtOHE) was prepared by dissolving ground basideocarp in 46% ethanol, at room temperature (± 25°C). The extract was centrifuged at 3,000 × g for 5 min, and the supernatant was lyophilized. The lyophilized was resuspended in DMEM, submitted to ultrafiltration in 0.2 μm pore size membrane, and stored at -20°C.

### Polysaccharide extraction and purification

The polysaccharide from *Lentinula edodes *(LeP) was isolated as described by Gonzaga et al. [19]. Briefly, dried mushroom was dissolved at 5% (w/w) in distilled water at 100°C during 5 h. The suspension was centrifuged and clear colorless extract was neutralized to pH 7.0 with 0.1 N NaOH. One percent NaCl was added and the extract submitted to polysaccharides precipitation with ethanol (5 vol ethanol:1 vol extract). After hydrogen peroxide/ethanol treatment, precipitate was resubmitted to ethanol extraction. The precipitate washed with ethanol and acetone was dried at 40°C, dissolved in distilled water, clarified by centrifugation and lyophilized.

### Cytotoxic assay

The cytotoxicity of the test substances was performed by dimethylthiazolyldiphenyl tetrazolium bromide (MTT) kit (**). HEp-2 cells were grown in 96-well microplates and treated with varying concentrations of AqE (0.1-100 mg/ml), EtOHE (0.1-40 mg/ml) and LeP (0.25-6.0 mg/ml). After 72 h incubation, the test was carried out according to the manufacturer's recommendation. Under the same conditions, cells without treatment were used as control. The 50% cytotoxic concentration (CC_50_) was determined as the concentration capable of reducing the optical density by 50% in comparison with the control. The CC_50 _was calculated by linear regression analysis of the dose-response curves generated.

### Plaque reduction assay

The antiviral activity by plaque reduction assay was done according to Melo et al. [20] and used throughout. Briefly, HEp-2 cells were cultivated in 24-well plates at 37°C in 5% of CO_2_. After complete confluence, the cells were infected and treated with the substances accordingly. Cell cultures were overlaid with nutrient agarose and supplemented with antibiotic. For PV-1 experiments, nutrient agarose was added of 25 mM MgCl_2_. The plates were incubated inverted at 37°C in 5% CO_2_, for 48 h. The cells were fixed with 20% formalin, stained with 0.5% crystal violet, after removal of the nutrient agarose layer. Concomitantly, mock-infected cells were used as control. The percentage of viral inhibition (% V.I.) was calculated by the formula: % V.I. = [1 - (number of plaques in test/number of plaques in virus control)] × 100.

The 50% inhibitory concentration (IC_50_) was determined as the concentration capable of reducing 50% the number of plaques forming units (PFU) in relation to the controls. The IC_50 _was determined by linear regression analysis of the curves of viral inhibition, for each treatment.

The selectivity index (SI) was calculated as the ratio of CC_50 _and IC_50_.

Strains of PV-1 and BoHV-1 were submitted to the treatment with 1,000 U/ml and 10,000 U/ml human alfa-2 interferon (Meizler Com. Intern. SA, Brazil), respectively.

### Virucidal activity

To evaluate the direct effect of the substances on viral particles, at varying concentrations, 10^6 ^PFU/ml of PV-1 and 10^5 ^PFU/ml of BoHV-1 were mixed with equal volumes of AqE (3.1, 6.3, 12.5 and 25 mg/ml), EtOHE (0.375, 0.75, 1.5 and 3 mg/ml) and LeP (0.025, 0.05, 0.1 and 0.2 mg/ml) for 1 h at 37°C and inoculated in cell cultures.

### Time-of-addition assay

The evaluation of the time-of-addition effect of the substances, at varying concentrations, was done as in Yang et al. [21]. Cells cultivated in 24-well plates were treated with concentrations of AqE (3.1, 6.3, 12.5 and 25 mg/ml), EtOHE (0.375, 0.75, 1.5 and 3 mg/ml) and LeP (0.025, 0.05, 0.1 and 0.2 mg/ml), before (-1 h and -2 h), during (0 h) and after (1 h and 2 h) infection.

### Viral adsorption assay

The inhibition of the viral adsorption was carried out according to Zhu et al. [22]. Briefly, the cells cultivated in 24-well plates were infected with viral strains in the presence of the AqE (3.1, 6.3, 12.5 and 25 mg/ml), EtOHE (0.375, 0.75, 1.5 and 3 mg/ml) and LeP (0.025, 0.05, 0.1 and 0.2 mg/ml), and incubated at 4°C in 5% CO_2 _for one h. The cells were washed twice with PBS, and plaque reduction assay was performed after 48 h.

### Statistics

The data were analyzed by ANOVA followed by Student's *t*-test. Values were considered significant to *p ≤ *0.05. All experiments were performed in triplicate.

## Results

**Figure 1:**
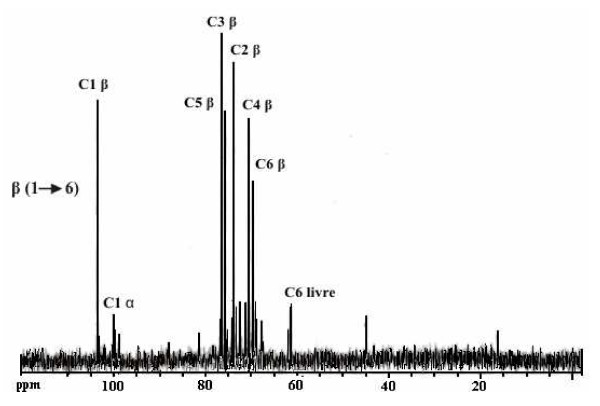
**^13^C NMR spectrum from polysaccharide isolated from *Lentinula edodes***.

The polysaccharide isolated from *Lentinula edodes *contains high levels of β-D-glucan. The ^13^C NMR spectrum is shown in Figure 1 and β(1→6) and α(1→4) glucans configurations were identified in the spectrum. The chemical displacements characteristics of the carbons C1 to C6 of the glycosidic ring are presented in Table 1.

**Table 1:** Chemical displacements characteristics of β(1→6) and α(1→4) glucans present in the purified polysaccharides isolated from *Lentinula edodes*

Configurations	Chemical displacements (ppm)					
	
	C1	C2	C3	C4	C5	C6
β(1→6)	103.5	73.7	75.6	70.4	75.6	69.7

α(1→4)	99.9	72.4	76.4	81.3	71.1	61.6

The cytotoxicity of the substances tested resulted in CC_50 _for the AqE, EtOHE and LeP of 74.0 mg/ml, 25.8 mg/ml and 4.0 mg/ml, respectively (Table 2).

**Table 2:** Antiviral activity of aqueous extract (AqE), ethanol extract (EtOHE) and polysaccharide (LeP) of *Lentinula edodes *for poliovirus and bovine herpesvirus, monitored by plaque assay

Substances	CC_50_^a^	PV-1		BHV-1	
		
		IC_50_^b^	SI^c^	IC_50_	SI
AqE	74.0	12.7	5.82	8.2	9.02

EtOHE	25.8	1.30	19.85	2.13	12.11

LeP	> 4.0	0.19	> 21.33	0.1	> 39.21

^a ^Fifty percent cytotoxic concentration (mg/ml)

^b ^Fifty percent inhibitory concentration (mg/ml)

^c ^Selectivity index

**Figure 2:**
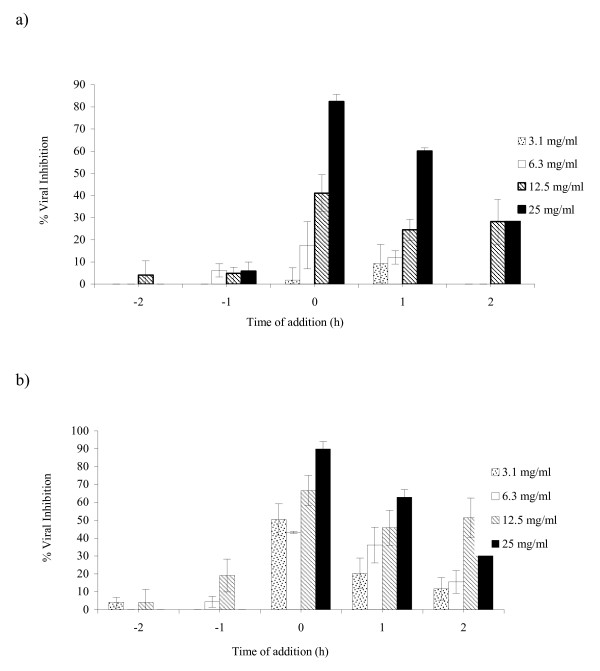
**Effect of *Lentinula edodes *aqueous extract (AqE) on poliovirus (a) and bovine herpesvirus (b) replication, monitored by plaque reduction assay in HEp-2 cells**. The extract was utilized in the indicated concentrations before (-1 and -2), during (0) and after infection (1 and 2). The experiments were carried out in triplicate, and the percent of the inhibition is represented with the respective standard deviations.

The results of antiviral activity for the AqE at different times of infection for PV-1 are shown in Figure 2a. When the AqE was added, one or two hours before infection (-1 h and -2 h), at the highest concentration tested (25 mg/ml), there was an inhibition of 5.8% and zero%, respectively. The addition of the extract at the concentrations of 3.1, 6.3, 12.5 and 25 mg/ml, at the moment of infection (0 h), resulted in a viral inhibition of 1.8, 17.5, 41.1 and 82.5%, respectively. However, for the time-of-addition of one hour post-infection (1 h), at the same concentrations, the percentages of inhibition were 9.2, 12.1, 24.5 and 60.2%, respectively. With the time-of-addition of two hours post-infection (2 h), the concentrations of 3.1 mg/ml and 6.3 mg/ml were not effective, but, the concentrations of 12.5 mg/ml and 25 mg/ml inhibited the replication of PV-1 by 28.2 and 49.4%, respectively.

The results of the tests for virucidal activity and the inhibition of adsorption showed that the AqE inhibited the replication of PV-1 by 38.3% and 19.0%, respectively, at the highest concentration tested. Figure 2b shows the results of the antiviral activity of AqE in the replication of BoHV-1. When the cells were treated with a concentration of 12.5 mg/ml, the highest percentage of viral inhibition obtained was 19.1 and 4.2%, for the times -1 h and -2 h before the infection, respectively. At the time 0 h, for the concentrations of 3.1, 6.3, 12.5 and 25 mg/ml there was inhibition of 50.4, 43.3, 66.7 and 89.9%, respectively. However, for post-infection treatments, at the same concentrations, there was inhibition of 20.4, 36.1, 45.8 and 63.0% for the time 1 h and 11.7, 15.5, 51.5 and 75.7% for the time 2 h.

For virucidal and inhibition of adsorption activities, there was a low inhibitory effect for the replication of BoHV-1 (32.1% and zero%, respectively) at the highest concentration of AqE.

**Figure 3:**
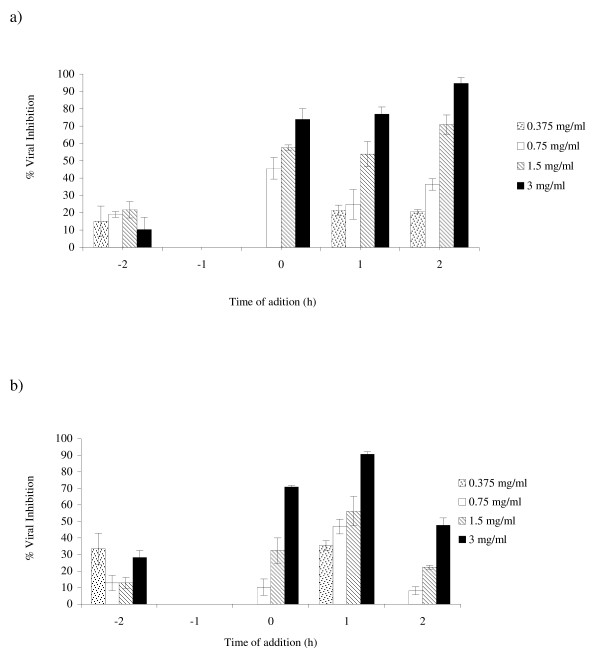
**Effect of *Lentinula edodes *ethanol extract (EtOHE) on poliovirus (a) and bovine herpesvirus (b) replication, monitored by plaque reduction assay in HEp-2 cells**. The extract was utilized in the indicated concentrations before (-1 and -2), during (0) and after infection (1 and 2). The experiments were carried out in triplicate, and the percent of the inhibition is represented with the respective standard deviations.

The results of the EtOHE on the replication of PV-1 and BoHV-1 are shown in Figure 3. For PV-1, at concentrations of 0.375, 0.75, 1.5 and 3 mg/ml, the extract inhibited replication of the virus by zero, 45.7, 56.5 and 73.9%, respectively, at time 0 h of infection (Figure 3a). When the extract was added at the times 1 h and 2 h, at the indicated concentrations, the percentages of inhibition were 21.4, 24.8, 53.9 and 76.9%, and 20.7, 36.3, 70.8 and 94.6%, respectively. However, treatments at times -1 h and -2 h, showed no significant effect, with the highest inhibition of 21.7%. In the replication of BoHV-1 (Figure 3b), at the same concentrations, the extract showed inhibition of zero, 10.2, 32.4 and 70.8%, respectively, for the time 0 h. Treatments at times 1 h and 2 h resulted in inhibition of 35.4, 46.9, 56.3 and 90.6%, and, zero, 8.1, 22.3 and 47.6%, respectively. The extract did not show any virucidal activity or inhibition of viral adsorption either for both virus strains.

**Figure 4:**
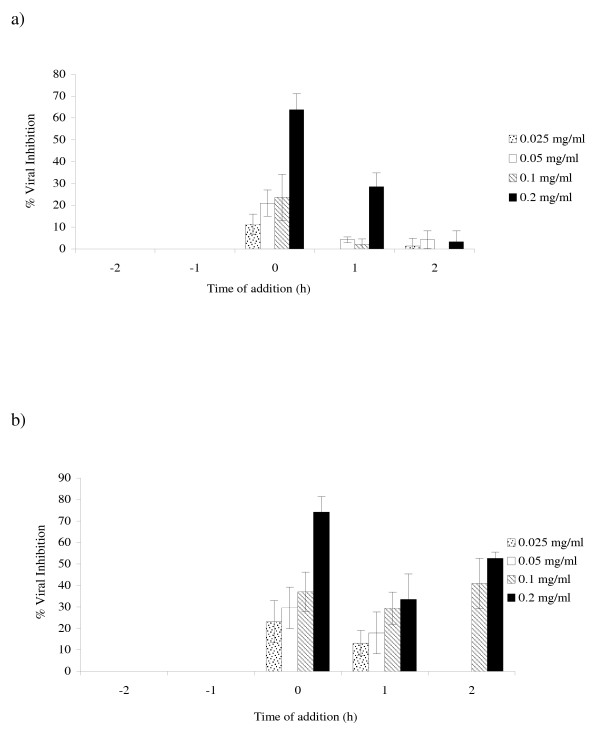
**Effect of *Lentinula edodes *polysaccharide (LeP) on poliovirus (a) and bovine herpesvirus (b) replication, monitored by plaque reduction assay in HEp-2 cells**. The extract was utilized in the indicated concentrations before (-1 and -2), during (0) and after infection (1 and 2). The experiments were carried out in triplicate, and the percent of the inhibition is represented with the respective standard deviations.

The antiviral activity of LeP on the replication of PV-1 and BoHV-1 are shown in Figure 4. For PV-1, at the concentrations of 0.025, 0.05, 0.1 and 0.2 mg/ml, LeP inhibited viral replication by 11.2, 21.0, 23.6 and 63.8%, respectively, at time 0 h of infection (Figure 4a). When the polysaccharide was added at time 1 h, at the same concentrations, the percentages of inhibition were zero, 4.2, 2.0 and 28.5%, respectively. However, for the treatments at the times 2 h, -1 h and -2 h, no effect was observed. In the replication of BoHV-1, at the same concentrations, LeP showed inhibition of 23.1, 29.6, 37.1 and 74.2%, respectively, for the time 0 h (Figure 4b). Treatments at the times 1 h and 2 h showed inhibition of 13.1, 17.9, 29.4 and 33.5%, and, zero, zero, 40.9 and 52.7%, respectively.

The LeP virucidal activity on the PV-1 replication, at the same concentrations, showed inhibition of 14.5, 24.4, 28.5 and 34.3%. However, LeP neither demonstrated virucidal activity for BoHV-1 nor inhibition of viral adsorption for both virus strains.

The IC_50 _and the respective SI for AqE, EtOHE and LeP, calculated for treatment at time 0 h of the infection, are shown in Table 2.

Interferon alpha-2, used as positive control, inhibited the replication of PV-1 and BoHV-1 by 100% at concentrations of 1,000 U/ml and 10,000 U/ml, respectively.

## Discussion

In this work, the aqueous and ethanol extracts, and, polysaccharides of the fruiting body of *Lentinula edodes *were evaluated for antiviral activity.

The results demonstrated that both extracts, as well as, LeP, inhibited the replication of PV-1 and BoHV-1. AqE inhibited PV-1 and BoHV-1 in a dose-dependent curve and the highest percentages of inhibition were obtained at time 0 h of infection. No significant inhibitory effect was observed when the extract was added at -1 h and -2 h of the infection and for the inhibition of adsorption, for both virus. This may suggest that AqE did not influence significantly the specific binding of both virus to cell receptors. AqE did not show virucidal activity, and therefore, did not affect virus particle directly either. Sorimachi et al. [23] demonstrated that fractions of the aqueous extract of *Agaricus blazei *mycelium inhibited significantly the CPE of western equine encephalitis (WEE) virus, herpes simplex virus and poliovirus in Vero cells. This effect was observed after infection demonstrating that extract of basidiomycetes may contain inhibitory compounds for the initial phases of replication at least for herpesvirus and enterovirus. This finding strengthens the activity that we demonstrated of AqE on PV-1 and BoHV-1 replication.

EtOHE was effective in inhibiting the replication of both viruses. The greatest percent of viral inhibition was demonstrated when the extract was added post-infection, 94.6% (2 h) and 90.6% (1 h) for PV-1 and BoHV-1, respectively. It is likely, therefore, that EtOHE acted on the initial steps of the replication, considering that AqE did not show virucidal activity or inhibition of viral adsorption either. Awadh Ali et al. [24] demonstrated the presence of antiviral activity in ethanol extracts of the fruiting body and mycelium of *Inonotus hispidus *against influenza virus A and B, attributing the effect to phenolic compounds. Concerning the relevance of glucan in biological activities it was demonstrated that LeP contains higher levels of β-D-glucan in comparison with an *Agaricus blazei *isolate [19]. In our study, the pronounced peak representative of anomeric carbon in β(1→6) configuration is evidence of greater concentration of glucan in relation to that of the α configuration. Many studies disclose β(1→3) configuration for the isolated glucan from *Lentinus *edodes [25,26], therefore, being different to the one disclosed in this study. Certainly, the characteristics of the cultivation region, such as climate and growth conditions, justify such behaviour. LeP, a β-glucan-protein with a predominance of β-1-6, also showed a dose-dependent inhibitory effect on BoHV-1 and PV-1, although less pronounced, nevertheless, with a higher SI and lower IC_50_, compared to AqE and EtOHE extracts. The antiviral activity of extracts isolated from the basidiomycetes seems to be mostly attributed to the presence of polysaccharides. The anionic feature of the molecules can interfere with early stages of viral replication [27,28] and sulfated polysaccharides demonstrate higher antiviral activity on enveloped virus [29].

## Conclusions

In conclusion, we suggest that AqE, EtOHE and LeP act on the initial processes of PV-1 and BoHV-1 replication. The extracts and the polysaccharide could be considered as a source of potential antiviral substances. However, further study is necessary to better understanding of the step(s) of viral replication where inhibition occurs.

## Competing interests

The authors declare that they have no competing interests.

## Authors' contributions

VPR participated in the studies on cytotoxicity and antiviral activity of the polysaccharide and extracts from *Lentinula edodes *and prepared the draft of the manuscript. KAY prepared the extracts and fractions and participated in the studies on antiviral activity. NMPSR and SAS participated in the extraction and purification of the polysaccharide, and participated in the manuscript. LDPM was responsible for shiitake cultivation and preparation of the extracts. RECL and CN were responsible for the design and coordination of the work, data analysis and manuscript writing and revision. All authors read and approved the final manuscript.

## References

[B1] De Clercq E Field HJ Antiviral prodrugs--the development of successful prodrug strategies for antiviral chemotherapy Br J Pharmacol 2006 147 1 11 10.1038/sj.bjp.0706446 16284630 PMC1615839

[B2] Jones PS Strategies for antiviral drug discovery Antivir Chem Chemother 1998 9 283 302 9875408

[B3] Strohl WR The role of natural products in a modern drug discovery program Drug Discov Today 2000 5 39 41 10.1016/S1359-6446(99)01443-9 10652450

[B4] Jiang T Luo S Chen Q Shen L Ying T Effect of integrated application of gamma irradiation and modified atmosphere packaging on physicochemical and microbiological properties of shiitake mushroom (*Lentinus edode*) Food Chem 2010 122 761 767 10.1016/j.foodchem.2010.03.050

[B5] Sugui MM De Lima PLA Delmanto RD Da Eira AF Salvadori DMF Ribeiro LR Antimutagenic effect of *Lentinula edode *(BERK.) Pegler mushroom and possible variation among lineages Food Chem Toxicol 2003 41 555 560 10.1016/S0278-6915(02)00306-X 12615128

[B6] Kupfahl C Geginat G Hof H Lentinan has a stimulatory effect on innate and adaptive immunity against murine *Listeria monocytogene *infection Int Immunopharmacol 2006 6 686 696 10.1016/j.intimp.2005.10.008 16504933

[B7] Suzuki M Takatsuki F Maeda YY Hamuro J Chihara G Antitumor and immunological activity of Lentinan in comparision with LPS Int J Immunopharmacol 1994 16 463 468 10.1016/0192-0561(94)90037-X 7927994

[B8] Maruyama S Sukekawa Y Kaneko Y Fujimoto S Anti-tumor activities of lentinan and micellapist in tumor-bearing mice Gan To Kagaku Ryoho 2006 33 1726 1729 17212088

[B9] Zhang L Li X Xu X Zeng F Correlation between antitumor activity, molecular weight, and conformation of lentinan Carbohydr Res 2005 340 1515 1521 10.1016/j.carres.2005.02.032 15882854

[B10] Jong SC Birmingham M Medicinal and therapeutic value of the shiitake mushroom Adv Appl Microbiol 1993 39 153 184 8213304 10.1016/s0065-2164(08)70595-1

[B11] Markova N Kussovski V Drandarska I Nikolaeva S Georgieva N Radoucheva T Protective activity of Lentinan in experimental tuberculosis Int Immunopharmacol 2003 3 1557 1562 10.1016/S1567-5769(03)00178-4 12946453

[B12] Kaneko Y Chihara G Friedman H et al Potentiation of host resistance against microbial infections by Lentinan and its related polysaccharides Microbial Infections 1992 New York: Plenum 201 206 10.1007/978-1-4615-3434-1_21 1414595

[B13] Sasaki SH Linhares REC Nozawa CM Montalván R Paccola-Meirelles LD Strains of *Lentinula edode *suppress growth of phytopathogenic fungi and inhibit Alagoas serotype of vesicular stomatitis virus Braz J Microbiol 2001 32 52 55 10.1590/S1517-83822001000100012

[B14] Wang S Welte T Fang H Oral Administration of Active Hexose Correlated Compound Enhances Host Resistance to West Nile Encephalitis in Mice J Nutr 2009 139 598 602 10.3945/jn.108.100297 19141700 PMC2646222

[B15] Racaniello VR One hundred years of poliovirus pathogenesis Virology 2006 344 9 16 10.1016/j.virol.2005.09.015 16364730

[B16] World Health Organization (WHO) Global polio eradication initiative http://www.polioeradication.org/Dataandmonitoring/Poliothisweek.aspx. Accessed 26 dec 2011. PMC2627439 16917643

[B17] Jones C Geiser V Henderson G Functional analysis of bovine herpesvirus 1 (BHV-1) genes expressed during latency Vet Microbiol 2006 113 199 210 10.1016/j.vetmic.2005.11.009 16352404

[B18] Hurk SDL Loehr BI Babiuk LA Immunization of livestock with DNA vaccines: current studies and future prospects Vaccine 2001 19 2474 2479 10.1016/S0264-410X(00)00476-X 11257380

[B19] Gonzaga MLC Ricardo NMPS Heatley F Soares SA Isolation and characterization of polysaccharides from *Agaricus blaze *Murill Carbohydr Polym 2005 60 43 49 10.1016/j.carbpol.2004.11.022

[B20] Melo FL Benati FJ Roman WA Jr Mello JCP Nozawa C Linhares REC The in vitro antiviral activity of an aliphatic nitrocompound from *Heteropteris aphrodisiac* Microbiol Res 2008 163 136 139 10.1016/j.micres.2006.03.011 16735108

[B21] Yang CM Cheng HY Lin TC Chiang LC Lin CC Acetone, ethanol and methanol extracts of *Phyllanthus urinari *inhibit HSV-2 infection in vitro Antiviral Res 2005 67 24 30 10.1016/j.antiviral.2005.02.008 15885815

[B22] Zhu W Chiu LCM Ooi VEC Chan PKS Ang PO Jr Antiviral property and mode of action of a sulphated polysaccharide from *Sargassum paten *against herpes simplex virus type 2 Int J Antimicrob Agents 2004 24 81 85 10.1016/j.ijantimicag.2004.02.022 15325432

[B23] Sorimachi K Ikehara Y Maezato G Inhibition by *Agaricus blaze *Murrill fractions of cytopathic effect induced by Western Equine Encephalitis (WEE) Virus on VERO cells in vitro Biosci Biotechnol Biochem 2001 65 1645 1647 10.1271/bbb.65.1645 11515550

[B24] Awadh Ali NA Mothana RAA Lesnau A Pilgrim H Lindesquist U Antiviral activity of *Inonotus hispidu* Fitoterapia 2003 74 483 485 10.1016/S0367-326X(03)00119-9 12837367

[B25] Surenjav U Zhang L Xu X Zhang X Zeng F Effects of molecular structure on antitumor activities of (1 → 3)--d-glucans from different *Lentinus Edode* Carbohydr Polym 2006 63 97 104 10.1016/j.carbpol.2005.08.011

[B26] Zhang Y Li S Wang X Zhang L Cheung PCK Advances in lentinan: Isolation, structure, chain conformation and bioactivities Food Hydrocolloid 2011 25 196 206 10.1016/j.foodhyd.2010.02.001

[B27] Eo SK Kim YS Oh KW Lee CK Lee YN Han SS Mode of Antiviral Activity of Water Soluble Components Isolated from *Elfvingia applanat *on Vesicular Stomatitis Virus Arch Pharm Res 2001 24 74 78 10.1007/BF02976497 11235816

[B28] Kabanov A Sementsova AO Skarnovich MO Teplyakova TV Shishkina LN Sergeev NA Development of new effective antiinfluenza drugs based on extracts of basidiomycetes Int J Infect Dis 2010 14 (Suppl 1):e88

[B29] Damonte EB Matulewicz MC Cerezo AS Sulfated seaweed polysaccharides as antiviral agents Curr Med Chem 2004 11 2399 2419 15379705 10.2174/0929867043364504

